# Effects of crystallographic and geometric orientation on ion beam sputtering of gold nanorods

**DOI:** 10.1038/s41598-017-17424-9

**Published:** 2018-01-11

**Authors:** J. A. Hinks, F. Hibberd, K. Hattar, A. Ilinov, D. C. Bufford, F. Djurabekova, G. Greaves, A. Kuronen, S. E. Donnelly, K. Nordlund

**Affiliations:** 1 School of Computing and Engineering, University of Huddersfield, Queensgate, Huddersfield, HD1 3DH United Kingdom; 2 University of Leeds, Leeds, LS2 9JT United Kingdom; 3 Sandia National Laboratories, Albuquerque, New Mexico 87123 USA; 4 Department of Physics and Helsinki Institute of Physics, University of Helsinki, PO Box 43, FI-00014 Helsinki, Finland

## Abstract

Nanostructures may be exposed to irradiation during their manufacture, their engineering and whilst in-service. The consequences of such bombardment can be vastly different from those seen in the bulk. In this paper, we combine transmission electron microscopy with *in situ* ion irradiation with complementary computer modelling techniques to explore the physics governing the effects of 1.7 MeV Au ions on gold nanorods. Phenomena surrounding the sputtering and associated morphological changes caused by the ion irradiation have been explored. In both the experiments and the simulations, large variations in the sputter yields from individual nanorods were observed. These sputter yields have been shown to correlate with the strength of channelling directions close to the direction in which the ion beam was incident. Craters decorated by ejecta blankets were found to form due to cluster emission thus explaining the high sputter yields.

## Introduction

With the continued drive to miniaturise technology and to explore the opportunities presented by nanoscience, there comes the need to understand the behaviour of nanomaterials under the various conditions potentially experienced both during manufacture and whilst in-service. Ion irradiation has long been used as a processing technique to modify the properties of materials in the semiconductor industry^1,2^ and focussed ion beam milling is increasingly being used to engineer nanostructures for a range of applications^3^. Once deployed, nanotechnologies may experience displacing irradiation in a wide variety of situations including nuclear reactors^4^, extraterrestrial environments^5^ and medical applications^6–8^.

The phenomena of channelling and sputtering are of particular importance to nanostructures exposed to these conditions. Channelling has the potential to vastly dilute the density of energy deposition and to extend the range of ions in a material; as the scale of the target is reduced, this can result in a shift of the nuclear energy deposition peak outside the dimensions of a nanostructure. Sputtering can result in the loss of material^9^, modification of surfaces^10^ and changes in geometry^11^. In the proximity of a surface, an atomic collision cascade can cause the mass ejection of atoms via cluster emission^12,13^. Such events can result in significantly-enhanced sputter yields in nanostructures with their increased surface-area-to-volume ratios. However, the potential for channelling to increase the depth at which cascades occur can ameliorate such effects.

Cluster emission was first reported in 1958 by Honig for low-energy inert-gas ion irradiation of silver, germanium and germanium-silicon alloy surfaces^14^. In high-density targets, the range of a primary knock-on atom is reduced due to the large cross-section for nuclear collisions resulting in denser cascades^15^. Additionally, in metals with low melting temperatures there arises the possibility of molten flow in the region heated by the thermal spike^16^. Thus there has been particular interest in such effects during the ion irradiation of bulk and thin-film gold^17–23^ and, more recently, of nanostructures^24,25^.

Recently we reported on enhanced sputter yields exceeding 10^3^ atoms.ion^−1^ for gold nanorods irradiated with 80 keV Xe ions both observed *in situ* via transmission electron microscopy (TEM) and simulated using molecular dynamics^12,13^. It was demonstrated that the experimental observations could be explained by the explosive emission of clusters and the ways in which the geometry of the nanorods made this significantly-more likely compared to the flat surface of a bulk sample. However, not all the nanorods studied in that work exhibited the same degree of sputtering and this was tentatively attributed to channelling effects dictated by the crystallographic orientations of the individual nanorods relative to the incident ion beam.

In the current paper, the aim is to extend that work to explore the effects in the higher-energy regime of 1.7 MeV Au ion irradiation. Theoretically, in a direct head-on collision, a 1.7 MeV Au ion can transfer all of its energy to a gold atom in a nanorod compared to a maximum of 76.8 keV for an 80 keV Xe ion as used in our previous work^12,13^. Therefore, effects due to the emission of clusters of atoms caused by a single ion are expected to be significantly enhanced at this higher energy offering greater possibilities to explore the processes of crater formation, plastic deformation and enhanced sputtering.

## Results

### TEM with *In Situ* Ion Irradiation

**Figure 1:**
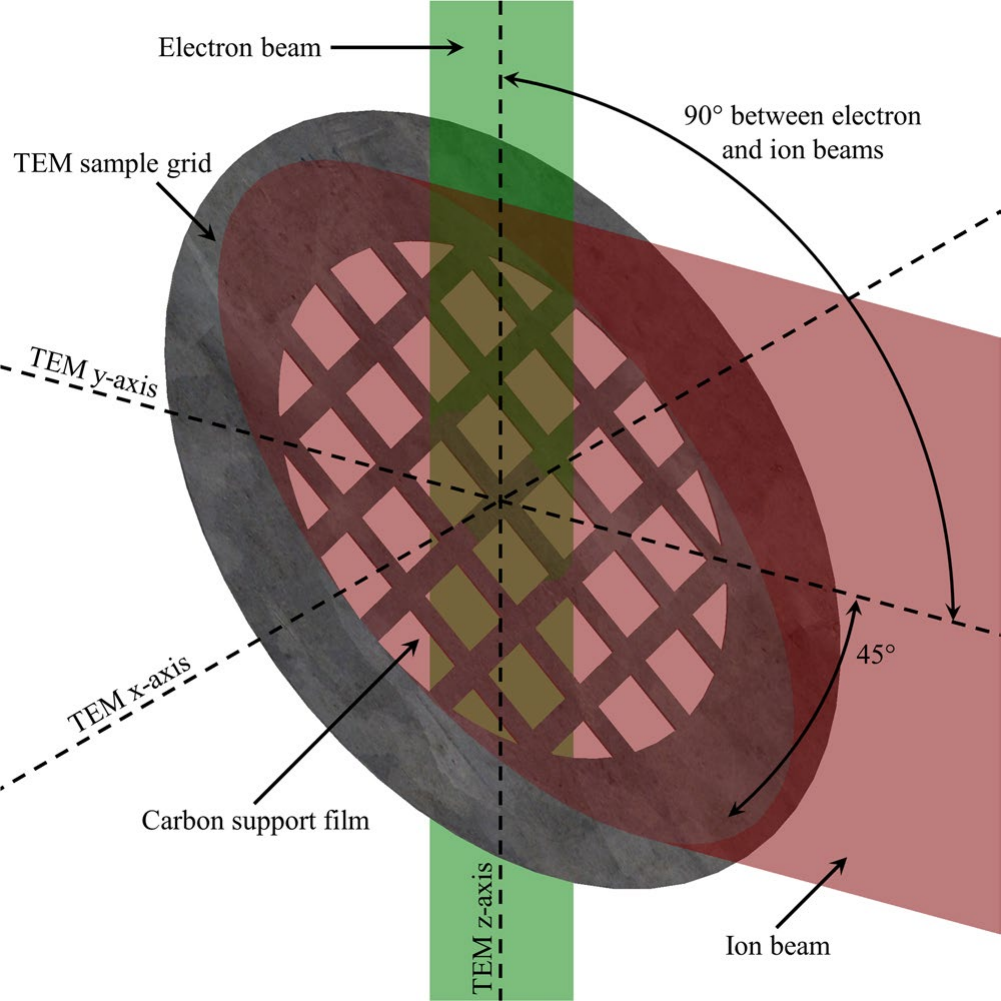
Schematic of the geometry of the TEM with *in situ* ion irradiation experiments performed using the I^3^TEM facility at Sandia National Laboratories. In the I^3^TEM facility, the electron and ion beams form an angle of 90° and the sample was tilted about the x-axis such that the support film formed an angle of 45° with both beams during irradiation.

The technique of TEM with *in situ* ion irradiation allows the direct observation of a sample whilst it undergoes dynamic microstructural evolution induced by the ion beam. Furthermore, electron diffraction data can be used to determine the crystallographic orientation of a nanostructure relative to the incident ion beam. Thus it is ideally suited to the explore ion beam sputtering and channelling effects in the individual gold nanorods studied for the work reported here. A schematic of the relative orientations of the electron beam, ion beam and TEM sample grid during the irradiations presented here is shown in Fig. 1.

**Figure 2:**
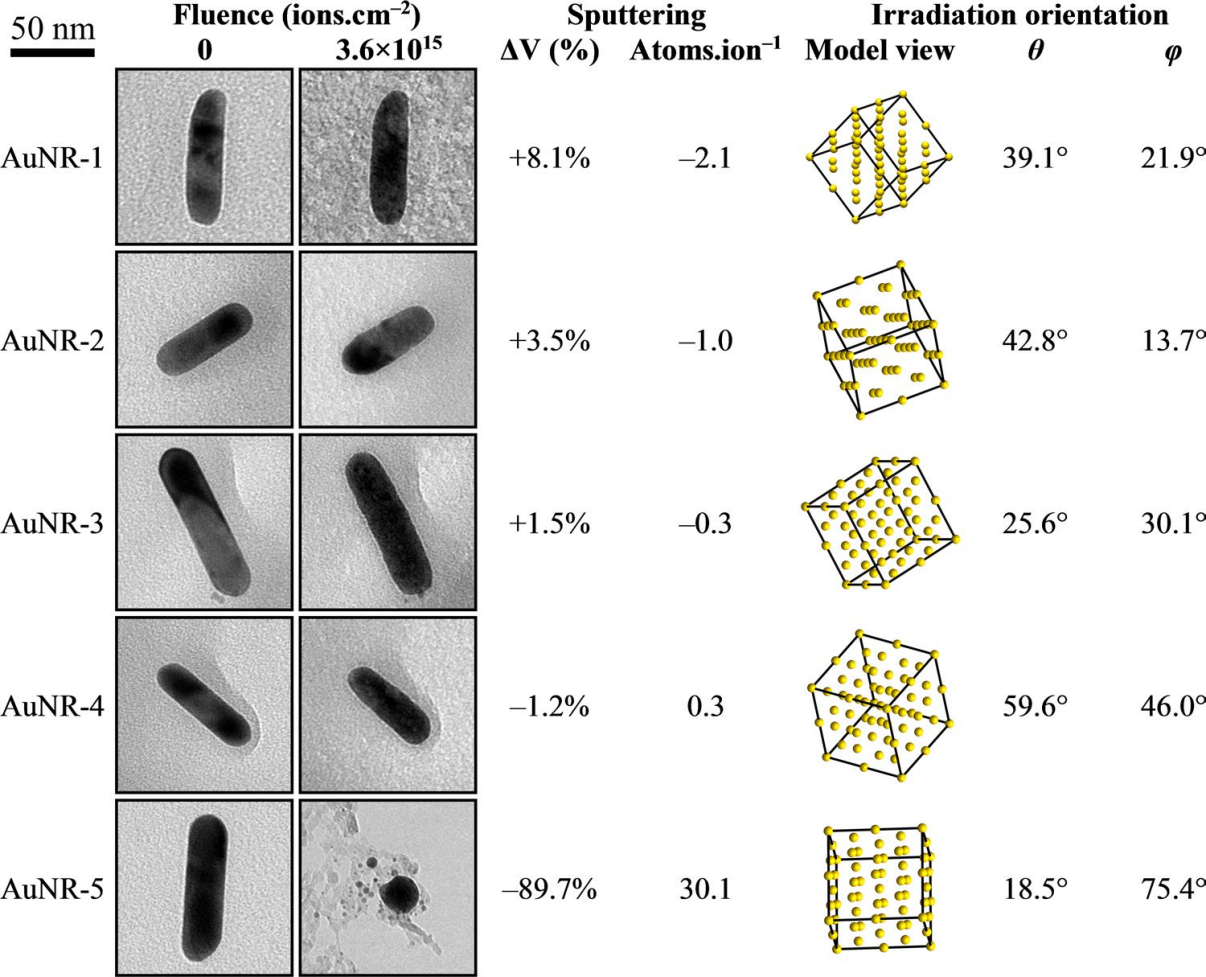
TEM micrographs and analysis of gold nanorods irradiated with 1.7 MeV Au ions for which the determination of the crystallographic orientation was possible from diffraction data. The micrographs in the figure have been rotated such that the ion beam is incident from the right in the image plane (i.e. the y-axis in the xy-plane of the TEM). The scale marker applies to all the micrographs in the figure.

Morphological changes were observed in all gold nanorods (referred to individually in this manuscript with a unique identifier composed of AuNR- (an abbreviation of gold (Au) NanoRod) and a numerical suffix) irradiated with 1.7 MeV Au ions. The magnitude of the sputter yield was found to vary by up to two orders of magnitude with most nanorods exhibiting a sputter yield towards the bottom of this range as summarised in Fig. 2. However, nanorods AuNR-5 and AuNR-6 were both observed to experience significantly-higher levels of sputtering under the same experimental conditions. Figure 2 also includes the crystallographic orientations of the nanorods illustrated both graphically by a visualisation of their crystal lattices and numerically by the angles between the ion beam and the [100] direction, *θ*, and between the ion beam and the [001] direction projected onto the (100) plane, *φ*. The TEM image series which show the evolution of nanorods AuNR-1 to AuNR-6 over 12 irradiation steps up to 3.6 × 10^15^ ions.cm^−2^ are presented in Supplementary Table S1.

**Figure 3:**
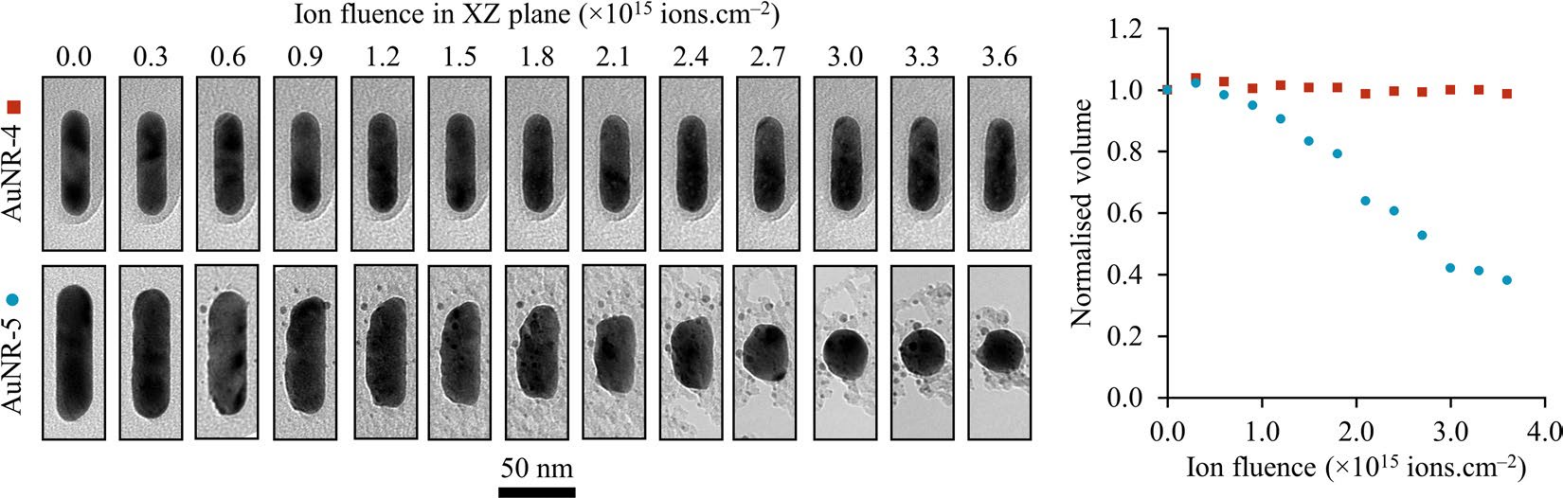
Comparison of two gold nanorods irradiated with 1.7 MeV Au ions and demonstrating significantly different sputtering effects for AuNR-4 (top row and red squares) and AuNR-5 (bottom row and blue circles). The micrographs have been rotated for presentation purposes; the directions from which the ion irradiation was incident for these two nanorods are shown in Fig. 2. The scale marker applies to all the micrographs in the figure.

The sputter rate for the nanorods was found to be relatively constant as a function of fluence; i.e. a nanorod which demonstrated a low sputter yield at low fluences would continue to do so and similarly a nanorod subject to greater sputtering effects in the early irradiations would continue to exhibit the same behaviour in subsequent steps. This is illustrated in Fig. 3 which compares nanorods AuNR-4 and AuNR-5. A slight shortening and broadening was experienced by nanorod AuNR-4 with a sputter yield of 0.3 atoms per incident ion. In contrast, nanorod AuNR-5 underwent more-significant loss of material due to sputtering with a yield almost two orders of magnitude higher at over 30 atoms per incident ion. The loss of the carbon support film around nanorod AuNR-5 at higher fluences which can be seen in Fig. 3 was due to greater electron beam exposure between ion irradiation steps as greater periods of time were spent on the TEM characterisation of this nanorod after the more-marked sputtering effects had been detected. The electron beam was not observed to have any effect on the nanorods themselves but was turned off during the ion irradiation (with the exception of AuNR-7) to preserve the carbon support film as discussed in the Methods section.

**Figure 4:**
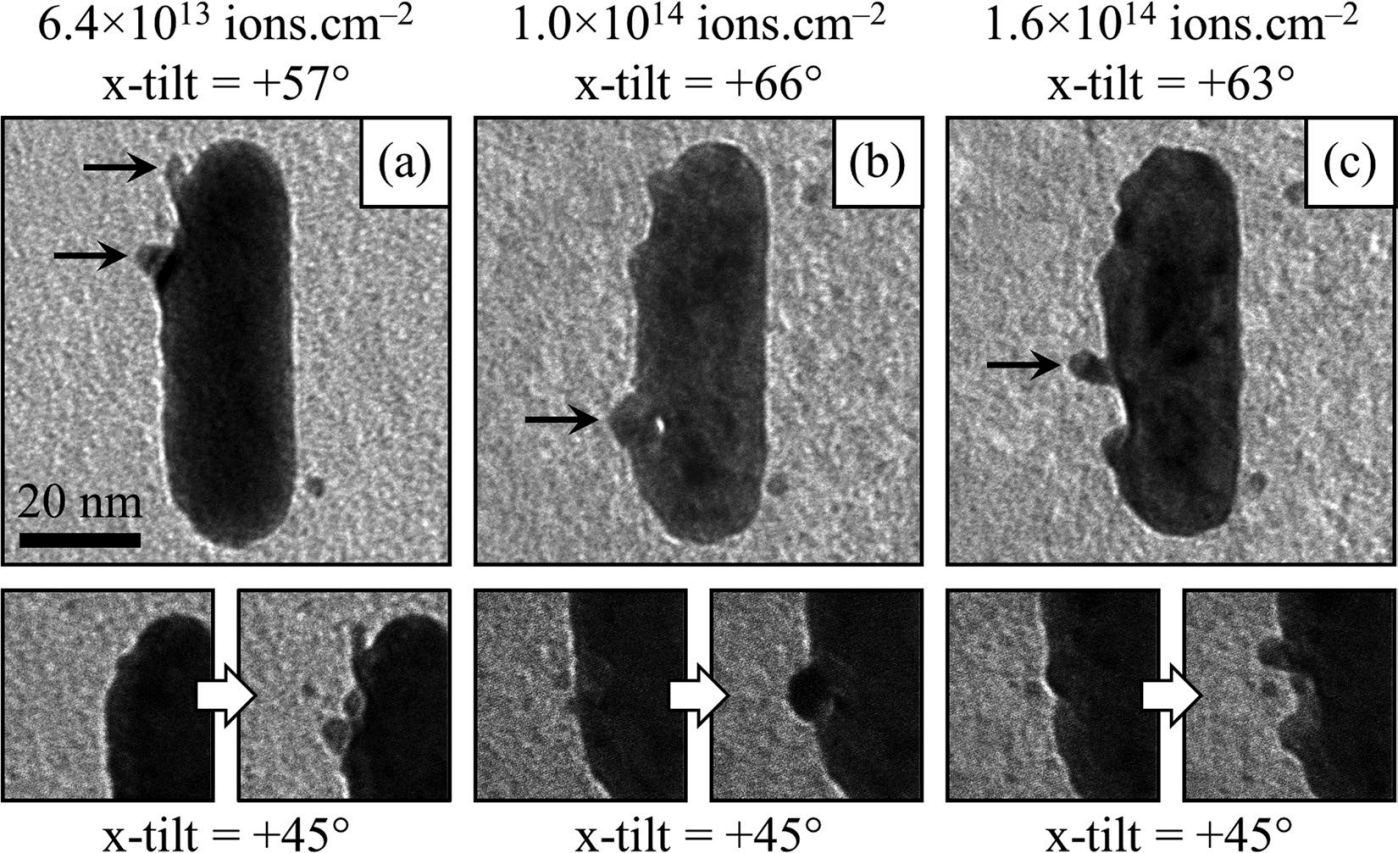
TEM micrographs of nanorod AuNR-7 demonstrating significant sputtering effects due to the 1.7 MeV Au ion irradiation. The top row of micrographs are selected views from the tilt series acquired in order to explore the morphological changes: (**a**) a crater surrounded by splash-like features; (**b**) an arch protruding from the surface; and (**c**) another splash-like feature at the boundary of a crater. The micrographs in the bottom row are frames taken from video captured during the ion irradiation (and hence at an x-tilt of +45° as described in the Methods section) showing the moments immediately before and after the formation of these features. The micrographs have been rotated such that the ion beam is incident from the right in the image plane (i.e. the y-axis in the xy-plane of the TEM). The scale marker in (**a**) applies to all the micrographs in the figure.

In addition to the gradual loss of material from the nanorods, various structures were observed to form during the irradiation which are consistent with material being quenched whilst undergoing flow processes as shown in Fig. 4: craters with diameters up to around 10 nm which were sometimes decorated with an ejecta blanket including splash-like features which extended outwards by up to around 5 nm; arches which protruded away from the nanorod surface; and small islands of gold on the carbon support film. The video clip in the Supplementary Information contains tilt series which capture the three-dimensional structures of these features.

### Channelling Calculations

**Figure 5:**
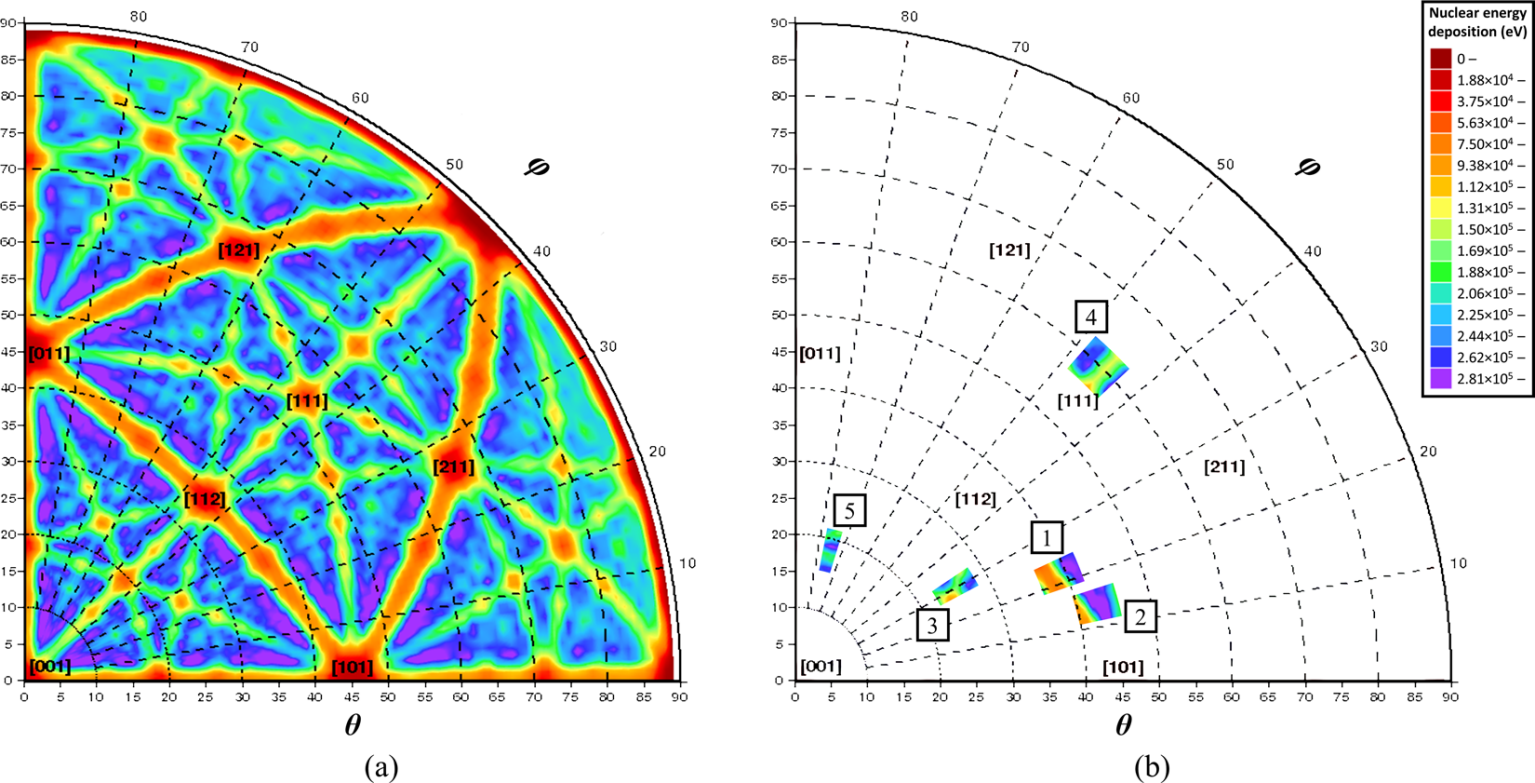
Channelling calculations performed with the MDRANGE computer code for 1.7 MeV Au ions incident on a 20 nm thick gold foil: (**a**) for 0 ≤ *θ* ≤ 89° and 0 ≤ *φ* ≤ 90° (results outside of these ranges are identical to those shown due to crystallographic symmetry); and (**b**) the ± 3° cones centred on the ion beam directions determined experimentally for nanorods AuNR-1 to AuNR-5. The colours indicate the nuclear energy deposition by the primary knock-on atoms with the Miller indices are shown for selected strong channelling directions. The colour scale in (**b**) applies to both plots.

The results of the MDRANGE channelling calculations for 1.7 MeV Au ions incident on a 20 nm thick gold foil are plotted in Fig. 5. They show that nuclear energy deposition can vary by more than one order of magnitude depending on the crystallographic direction; varying between about 10 keV for a <011> channel to a maximum of 300 keV in several non-channelling directions. The results of the MDRANGE calculations are available in the Supplementary Information.

### Molecular Dynamics Simulations

**Figure 6:**
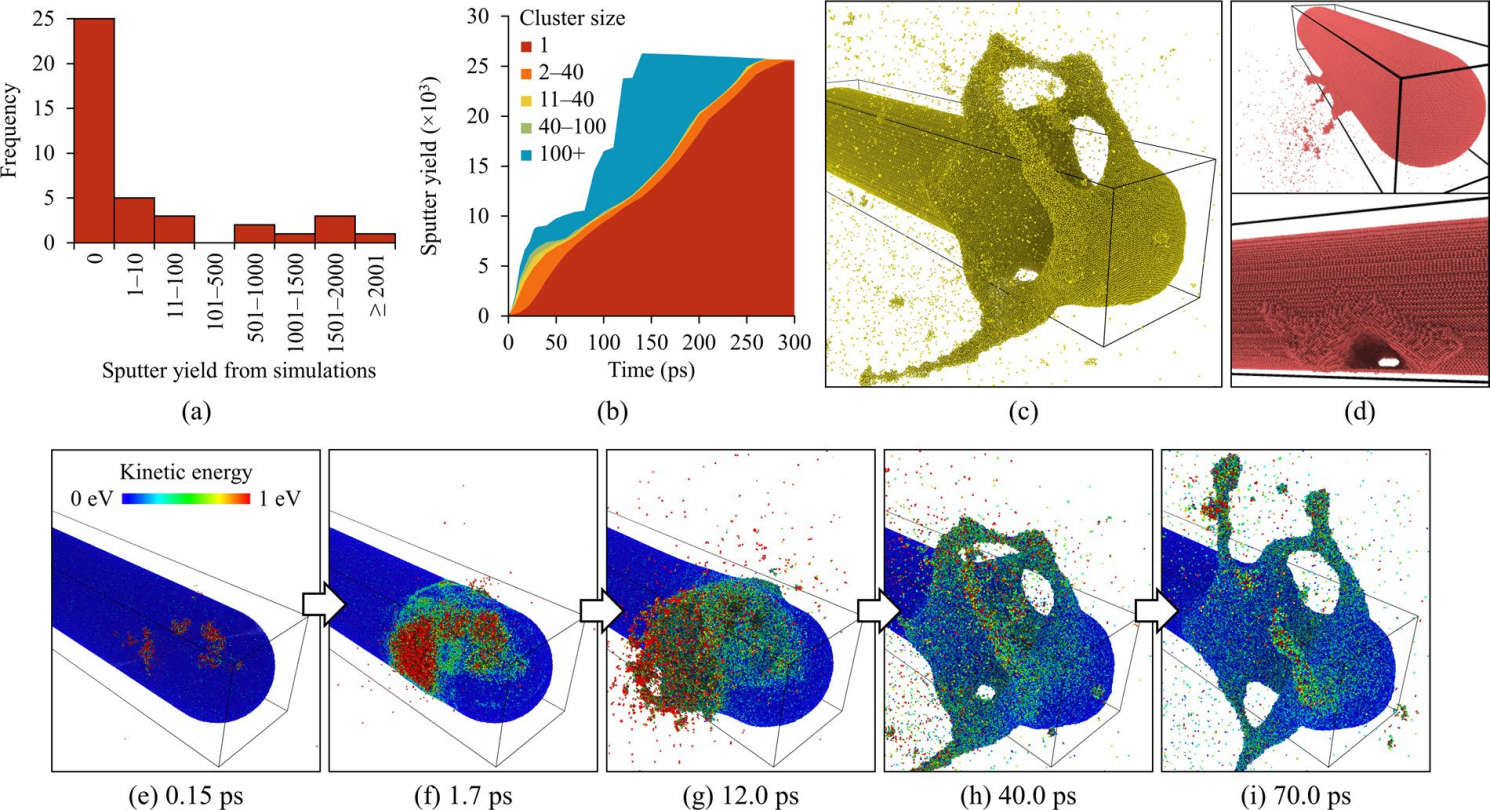
Results of the molecular dynamics simulations of 1.7 MeV atoms incident on a 100 × 20 × 20 nm spherocylindrical nanorods each containing 1.7 × 10^6^ gold atoms: (**a**) distribution of the calculated sputter yields; (**b**) contribution of different cluster sizes to the sputter yield as a function of time for the simulation shown in the visualisation in (**c**,**d**) visualisation of another simulation after 27 ps showing cluster emission due to an atomic collision cascade in the proximity of the surface and the end state showing a tunnel-like crater created by the ejection of material; (**e–i**) series of visualisations showing the evolution of an atomic collision cascade and distribution of kinetic energy over the first 70 ps of the simulation featured in the molecular dynamics video clip in the Supplementary Information.

Despite the selection of a non-channelling direction, the majority of the molecular dynamics simulations gave a sputter yield of zero. This was because in these cases the ion was transmitted through the nanorod without undergoing any strong nuclear collisions. A systematic analysis of MDRANGE calculations along the direction used for the simulations has demonstrated that this is to be expected (see Supplementary Information). However, a number of simulations also generated significant cascades sufficiently close to a surface to cause cluster emission resulting in sputter yields in excess of 10^3^ atoms in several cases as shown in Fig. 6(a). Examples of simulations featuring cluster emission and concomitantly-higher sputter yields are shown in Fig. 6(c–i) and the molecular dynamics video clip in the Supplementary Information. Although material was initially ejected as clusters of atoms, these were found to fragment such that almost all the sputtered material ended up in the form of individual atoms as illustrated in Fig. 6(b) which demonstrates how the vast majority of sputtered material was in the form of individual atoms after 300 ps in the simulation shown in Fig. 6(c). The average sputter yield across the 40 simulations performed for the current work was 254 atoms.

## Discussion

Sputtering of the gold nanorods was observed during irradiation with 1.7 MeV Au ions both in the experiments and the simulations. This occurred via both the continuous loss of material due to the sputtering of individual atoms but also in discrete events which resulted in mass ejections leaving behind craters on the nanorods. Molecular dynamics simulations have revealed the latter to be due to an atomic collision cascade near the surface of the nanorod causing the emission of a cluster of atoms. The experimentally-measured sputter yields varied from −2.1 to 30.1 whereas the molecular dynamics simulations gave an average value of 254. The vastly different sputter yields, varying by up to two orders of magnitude, measured in the experiments reported here under 1.7 MeV Au ion irradiation have similarly been observed in previous work^12,13^ using 80 keV Xe at the MIAMI facility^26^. Several explanations could account for the large range of sputter yields observed across the nanorods studied as discussed below.

**Figure 7:**
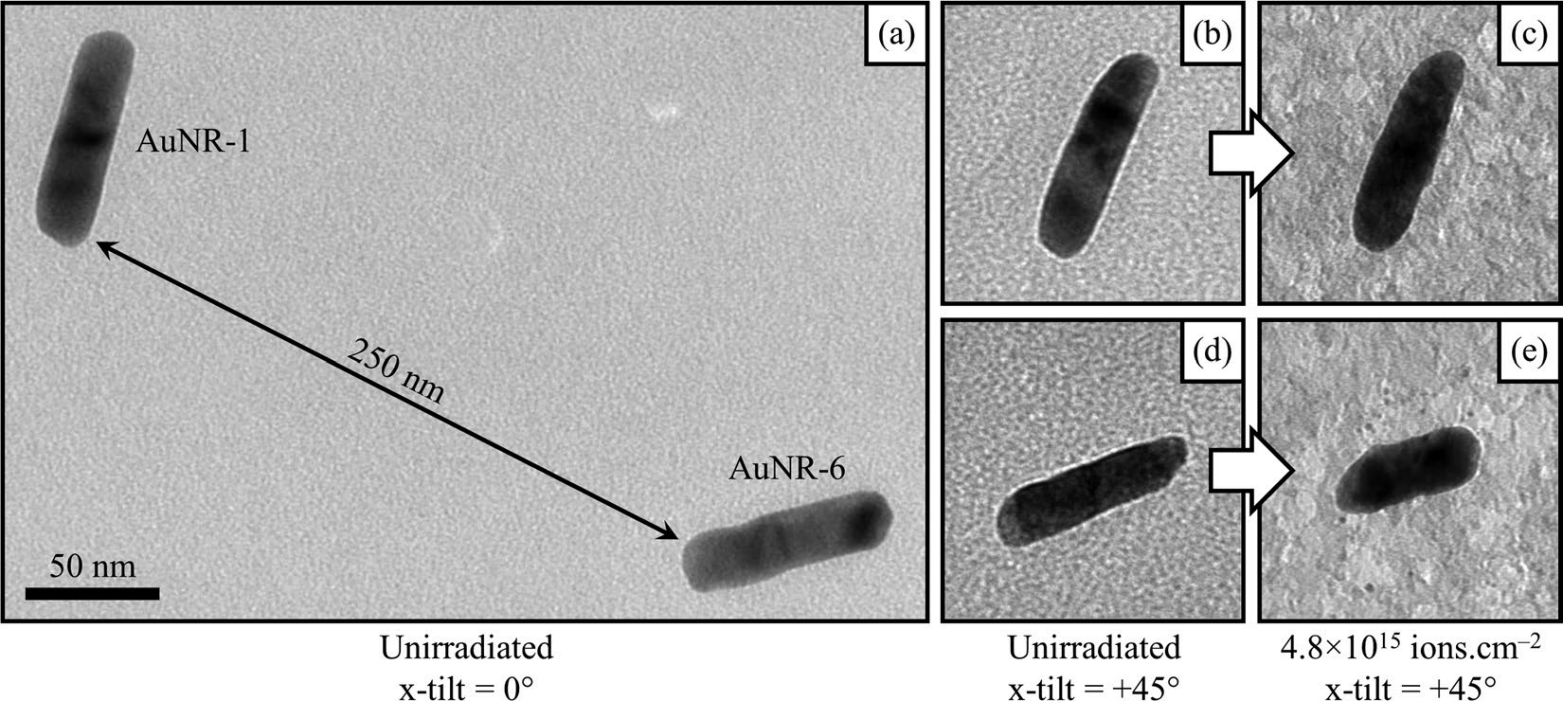
TEM micrographs demonstrating significantly-different levels of sputtering from neighbouring nanorods AuNR-1 and AuNR-6 irradiated with 1.7 MeV Au ions: (**a**) before irradiation at an x-tilt of 0° showing the separation of the nanorods to be on the order of 250 nm in the plane of the support film; (**b**) and (**d**) before irradiation at an x-tilt of +45°; and (**c**) and (**e**) after a fluence of 4.8 × 10^15^ ions.cm^–2^. The scale marker in (**a**) applies to all the micrographs in the figure.

Firstly, if undetected inhomogeneities were present in the ion beam then these could potentially cause differing effects across the irradiated area. Figure 7 shows nanorods AuNR-1 and AuNR-6 which were located on the carbon support film at a separation of 250 nm as observed at a TEM x-tilt of 0°. During the irradiation at a TEM x-tilt of 45°, that distance would have been even smaller when projected in the direction of the ion beam as illustrated in Fig. 1. These two nanorods demonstrated significantly-different sputter yields of −2.1 and 2.6 despite their close proximity suggesting that local variations in ion beam flux were not responsible. Ion beam shadowing effects could cause large differences in ion flux over very short distances but this can also be ruled out given the design of the tomography sample holder, the high degree of TEM x-tilt and the deliberate selection of nanorods distant from the mesh bars of the TEM grid.

Another important consideration is the geometric orientation of the nanorods relative to the incident ion beam. As the projected area presented by a nanorod to the ion beam is taken into account in the calculations of the sputter yields (see Supplementary Information for details of the calculation method), the importance of geometric orientation for the sputter yields reported here would be in the range of incidence angles made between the nanorod surface and the ion beam as sputtering is a strong function of angle^27^. As can be seen in Fig. 2, comparison of nanorods with similar orientations reveals markedly-different levels of sputtering leading to the conclusion that geometry is not the most important factor in determining yield. Most notable in this respect are nanorods AuNR-1 and AuNR-5 with sputter yields of −2.1 and 30.1, respectively.

As no other significant differences were observed between the nanorods, such as the presence of oxide or contamination layers, then the most likely candidate to account for the variations in sputter yield is their crystallographic orientations. Channelling effects are known to be able to significantly extend the range, and thus reduce the energy deposition, of ions^28^. The nuclear energy deposition peak for 1.7 MeV Au ions in gold is about 50 nm (see Supplementary Information for details of the calculation method) and therefore such effects could significantly reduce the number of nuclear collisions occurring within the nanorod volume. Indeed, it should be noted that in the study of the ion-irradiation-induced morphological changes presented in Fig. 4, the craters are all formed on the side of the nanorod facing away from the ion beam suggesting that any extension of the ion range could have significantly reduced the probability of these events occurring. The results of the channelling calculations performed using the MDRANGE computer code shown in Fig. 5(a) demonstrate the presence of channelling and non-channelling directions in which nuclear energy deposition can vary by a factor of 30. In Fig. 5(b), the ± 3° annular cones around the crystallographic directions of the incident ion beam for nanorods AuNR-1 to AuNR-5 are presented to visualise the results given in Table 1 for the average, $\langle {F}_{{D}_{n}}\rangle$, and minimum, ${F}_{{D}_{n,{\rm{\min }}}}$, nuclear energy deposition values. The experimentally measured sputter yields do not follow the calculated average values for nuclear energy deposition, 〈${F}_{{D}_{n}}$〉. However, with in the annular cones the directions with the lowest nuclear energy deposition values, ${F}_{{D}_{n,{\rm{\min }}}}$, do correlate with the sputter yields. Inspection of the regions of Fig. 5(b) corresponding to nanorods AuNR-1 to AuNR-5 reveals the presence of strong channelling directions in the proximity of nanorods AuNR-1 to AuNR-4 (which had relatively-low sputter yields) and an absence of such directions for AuNR-5 (which had the highest).

**Table 1:** Comparison of the experimentally-measured sputter yields with the MDRANGE nuclear energy deposition calculations for 1.7 MeV Au ions travelling through a 20 nm gold foil in the crystallographic directions determined to have been presented to the ion beam by the nanorods during the irradiations.

Nanorod	Sputter yield	〈${{\boldsymbol{F}}}_{{{\boldsymbol{D}}}_{{\boldsymbol{n}}}}$〉	${{\boldsymbol{F}}}_{{{\boldsymbol{D}}}_{{\boldsymbol{n}},{\bf{m}}{\bf{i}}{\bf{n}}}}$
	Atoms.ion^−1^	×10^5^ eV	×10^5^ eV
AuNR-1	−2.1	1.69	0.68
AuNR-2	−1.0	2.27	0.80
AuNR-3	−0.3	1.90	0.91
AuNR-4	0.3	1.78	0.87
AuNR-5	30.1	2.22	1.65

Experimental investigations into ion channelling often rotate single crystals in the beam path whilst monitoring a quantity such as sputter yield. Channelling manifests itself as a reduction in the measured yield over a small angular range when a channelling direction is traversed. This angular range is normally a fraction of a degree^29^ compared to the annular cones of a few degrees being considered here. It is possible that the combined error from the mechanical accuracy of the TEM x-tilt and from the diffractions conditions (i.e. ***s***_**g**_ ≠ 0), means that the orientations of the nanorods deviated from those expected sufficiently for the ion beam to have been incident along a direction closer to ${F}_{{D}_{n,{\rm{\min }}}}$. Another explanation for this discrepancy could be that the ions imparted sufficient momentum through nuclear collisions to induce small random rotations to the nanorods which then aligned them onto the nearest channelling direction; once on a channelling direction, the probability of further such collisions would be greatly reduced and thus the nanorods would tend to stay in this orientation. In the case of a highly-sputtered nanorod such as nanorod AuNR-5 for which ${F}_{{D}_{n,{\rm{\min }}}}/\langle {F}_{{D}_{n}}\rangle$ = 0.74, the driving force for this alignment to be maintained once reached would be much less than for nanorod AuNR-2 for which ${F}_{{D}_{n,{\rm{\min }}}}/\langle {F}_{{D}_{n}}\rangle$ = 0.35 and which demonstrated a relatively-low sputter yield.

Whilst the absence of an accessible channelling direction for AuNR-5 likely explains the large disparity between its sputter yield of 30.1 and the next-closest of 2.6 for nanorod AuNR-6, another possible difference could lie in the adhesive forces between the nanorods and the carbon support film. If a nanorod settles into an undulation or an area of contamination on the carbon support film then it is possible that the effective surface area of contact could be increased to such a degree that the ion beam cannot induce a rotation sufficient to access the orientation corresponding to ${F}_{{D}_{n,{\rm{\min }}}}$.

The difference between the experimentally-measured sputter yields and those from the molecular dynamics is expected as the simulations were performed deliberately on a non-channelling crystallographic direction. However, another explanation for the discrepancy may lie in the calculation of the volumes of the nanorods (see Supplementary Information for details of the calculation method). This procedure involved dividing a nanorod into a series of thin slices which are then treated as short cylinders assuming that the nanorods remain approximately circular in each cross section. Analysis of images from the tilt series taken of irradiated nanorods have shown this to be generally the case in terms of the overall geometry. However, this approach can overestimate the volume, and thus underestimate the sputter yield, in the presence of a large crater especially if the crater does not affect the projected shape of the nanorod in the TEM micrograph. Therefore, at low sputter yields it is possible for a negative sputter yield (i.e. an addition of atoms) to be calculated as can be seen in Fig. 2.

Comparison of the experimentally-observed ion-beam-induced features in Fig. 4 (and associated video in the Supplementary Information) with the structures modelled in the molecular dynamics simulations shown in Fig. 6 reveals some notable similarities. In both cases, craters on the surface of the nanorods were observed and were surrounded by material analogous to the ejecta blanket found around meteorite impact craters^30^. The similarity between the arch shown in Fig. 4(b) and the tunnel crater in Fig. 6(d) is also striking.

During the ion irradiation experiments, the carbon support films around the nanorods became decorated with islands of gold atoms due to sputter deposition. These can be seen in many of the micrographs shown in Figs 2, 3, 4 and 7. Similar islands have been reported for gold nanoparticles subjected to a variety of irradiation conditions and are hypothesized to form by different mechanisms. In the case of light keV-range ions, the energy transfer is sufficient to eject only individual atoms or small clusters^29^. Hence island formation must result from diffusion-based agglomeration processes in those situations. For heavier more-energetic ions such as 1.7 MeV Au ions, both sputtering of single atoms and cluster emission become possible^12^. In the current study, the islands were often observed to appear within a time period of less than one frame of video (equivalent to 0.07 s) concurrently with the formation of a crater as shown in the video frames in Fig. 4. Analysis of the evolution of the size distribution of ejected clusters as a function of time in the molecular dynamics simulations suggests that they disintegrate within a few 100 s of ps as illustrated in Fig. 6(b). However, in previous studies^31^ as well as in the simulations reported here, it has been shown that ejected clusters can travel distances comparable to the diameter of a nanorod before disintegrating so it is therefore plausible that these islands did indeed form via cluster deposition in at least some instances.

Complementary experimental and theoretical studies of 1.7 MeV Au ion irradiation of gold nanorods have produced new insights into the physics of ion-beam-induced sputtering of these nanostructures. Using TEM with *in situ* ion irradiation at the I^3^TEM facility at Sandia National Laboratories, large variations in the sputter yield have been observed between different nanorods. Calculations of nuclear energy deposition as a function of crystallographic orientation have revealed a correlation between the experimentally-measured sputter yields and the strength of the channelling directions found within annular cones of ± 3° of the ion irradiation direction. It is hypothesised that the ion beam may induce small rotations to the nanorods and if a channelling direction is reached then the probability of further tilting is vastly reduced and hence the nanorod will tend to remain thus orientated. Further experiments and modelling are required to explore the nanorod rotations which can be induced by ion irradiation and the plausibility of the proposed mechanism for preferential alignment along a channelling direction.

Sputter yields of up to 30 atoms.ion^−1^ (expected to be an underestimate) have been measured experimentally compared to 254 atoms for the non-channelling direction in the molecular dynamics simulations. Such high sputter yields are explained by the observation, in both the experiments and the simulations, of the ejection of clusters of atoms. The emission of such clusters produced craters decorated by ejecta blankets on the surface of the nanorod in both the experiments and the simulations. Moreover, in the case of the experiments, islands of deposited gold on the surrounding carbon support film were observed to appear within a timespan of a video frame. Simulations have shown that the clusters are expected to fragment after emission and therefore it is likely that the observed gold islands are due to both the arrival of single large clusters and the agglomeration of sputtered atoms moving across the surface of the support film.

From a fundamental perspective, these results give new insights into the atomistic processes at work during the ion irradiation of nanostructures. Previous work has looked at the effects of size and geometry on sputtering in systems such as silicon nanospheres^32^ and gold nanoparticles^33^. Our recent work has studied enhanced sputtering of gold nanorods due to cluster emission under 80 keV Xe ion irradiation and identified large differences in sputter yield between individual nanorods^12,13^. This has now been extended to the higher-energy regime of 1.7 MeV Au ions identifying channelling as the most likely explanation for the observed differences and capturing the more-pronounced formation of surface structures during cluster emission.

These results will be important for the development of nanotechnologies in terms of both their manufacture and modification using ion irradiation techniques and also for their deployment in environments in which they are exposed to irradiation. For example, gold nanoparticles are being studied as radiation sensitizers to improve the efficacy of proton beams for cancer treatment^6^. Understanding of the radiation response of nanoparticles, especially where it differs from that of bulk material, will help in the design of therapies using optimised combinations of irradiation conditions and nanoparticle composition, size and distribution. This work will be of even greater relevance to the use of such nanoparticles in heavy-ion therapies^7,8^ which are more likely to produce the effects reported here. In the area of nuclear energy, nanoparticles have been proposed for use in nanofluids to improve heat transfer efficiency^4^. These will be bombarded by neutrons in primary coolant loops and detailed understanding of the optional effects this may have will aid the development and deployment of this technology.

## Methods

### TEM with *In Situ* Ion Irradiation

Gold nanorods suspended in deionised water were obtained from Nanopartz Inc (product number A12-25-850-25) and deposited on carbon film supports on 400 mesh copper TEM grids. The nanorods were measured to be of diameter 20 nm with typical lengths in the range of 60–100 nm. The majority were found to be <001> orientated along their axes but were otherwise randomly orientated relative to the support grid due to the dispersive nature of the TEM sample preparation technique. Individual isolated nanorods distant from the mesh bars of the TEM grid were selected for the irradiation experiments to avoid shadowing and sputter deposition effects from the mesh bars and from other nanorods.

The I^3^TEM *in situ* ion irradiation facility at Sandia National Laboratories (NM, USA) combines a 6 MV tandem accelerator with a JEOL JEM-2100 TEM and is described in detail elsewhere^34^. The ion beam is orientated horizontally such that the electron beam, ion beam and x-axis of the TEM are all orthogonal to each other as shown in Fig. 1. For the experiments reported here, the samples were irradiated with 1.7 MeV Au^3+^ ions with a flux of 1.5 × 10^13^ ions.cm^–2^.s^−1^ at room temperature up to a fluence of either 3.6 × 10^15^ or 4.8 × 10^15^ ions.cm^−2^ in steps of 3.0 × 10^14^ ions.cm^−2^. All fluences are per unit area in the xz-plane of the TEM. The TEM was operated at 200 kV and the electron beam was not incident on the sample during ion irradiation. Prolonged electron beam exposure resulted in no observable changes to the gold nanorods. However, the carbon film did demonstrate roughening and ultimately decomposition under the electron beam; therefore, it was necessary to minimise electron beam exposure to preserve the integrity of the support.

Additional details of the TEM with *in situ* ion irradiation experimental techniques and associated calculations used for the current work are given in the Supplementary Information.

### Channelling Calculations

Nuclear energy deposition as a function of crystallographic direction was calculated for 1.7 MeV Au ion irradiation using molecular dynamics simulations with the recoil interaction approximation in the MDRANGE computer code^35^. A gold target was generated as a perfect FCC crystal with random thermal displacements of atoms corresponding to 300 K. The simulations were run using the ZBL^36^ and DMol^37^ interatomic-pair potentials but both were found to give essentially identical results; the results presented here were produced using the ZBL potential.

The simulations were carried out in the ranges of 0–89° for tilt, *θ*, and 0–90° for twist, *φ*, off the [001] direction in increments of 1° and 2°, respectively. For each *θ*, *φ* combination, 10^3^ ions were simulated and the average nuclear energy deposition was calculated over a distance of 20 nm in the target. A bilinear interpolation was used to achieve a smooth energy-deposition plot^38^. The results of these calculations (prior to interpolation) are included in the Supplementary Information.

### Molecular Dynamics Simulations

Spherocylindrical nanorods measuring 100 × 20 × 20 nm and containing 1.7 × 10^6^ gold atoms were simulated using molecular dynamics. They were then relaxed for 5 ps at 312 K using linear Berendsen temperature control^39^ and then rotated 13° about the major axis (i.e. *θ* = 13° and *φ* = 0° so as to be 13° off a {001}) to reduce the chance of channelling^40^.

Molecular dynamic simulations were run with the PARCAS code^41^ using the Foiles *et al*.^42^ implementation of the embedded atom model (EAM)^43,44^. The EAM reliably models surface energy, melting temperature and the liquid state^45^ which are essential to the modelling of ion impacts and sputtering in metals. This approach has previously demonstrated good agreement with experimental results on surface irradiation effects^12,13^; however, Samela *et al*.^46^ have highlighted some drawbacks and to help address these the EAM potential was combined with the universal Ziegler-Biersack-Littmark (ZBL) repulsive potential^36^. The outcome of an atomic collision cascade tends to be determined by the liquid zone^41^ which is not affected by the choice of repulsive potential and therefore any inaccuracies of the ZBL potential should not be significant here. In addition, ZBL electronic stopping was applied for all atoms in the system which had ≥ 5 eV of kinetic energy^35^.

A 1.7 MeV Au atom moving vertically downwards was then generated at a random position in a horizontal plane above the nanorod. Simulations were terminated if the incident atom transferred less than 170 eV to the nanorod for the purposes of computational efficiency by excluding events which would not result in any significant erosion. Although terminated early, such simulations were still included in the calculation of the average sputter yield of 254 atoms reported here. Visualisation and analysis of the results was performed using the Open Visualization Tool (OVITO) software^47^.

### Data Availability

The datasets generated during and/or analysed during the current study are available from the corresponding author on reasonable request.

## Electronic supplementary material

Supplementary Information

Video of TEM tilt series

Video of molecular dynamics simulation

MDRANGE channelling map data file
