# Supplementary material for: Effects of crystallographic and geometric orientation on ion beam sputtering of gold nanorods
Source: Sci Rep. 2018 Jan 11;8:512. doi: 10.1038/s41598-017-17424-9 (PMC5765137; doi:10.1038/s41598-017-17424-9)
Supplement: Supplementary file 1 — Supplementary Information [file 41598_2017_17424_MOESM1_ESM.pdf]

Effects of crystallographic and geometric orientation on ion beam sputtering of gold nanorods

J.A. Hinks[1], F. Hibberd[2], K. Hattar[3], A. Ilinov[4], D.C. Bufford[3], F. Djurabekova[4], G. Greaves[1], A. Kuronen[4], S.E. Donnelly[1] and K. Nordlund[4]

[1] School of Computing and Engineering, University of Huddersfield, Queensgate, Huddersfield, HD3 4FU, United Kingdom
[2] University of Leeds, Leeds, LS2 9JT, United Kingdom
[3] Sandia National Laboratories, Albuquerque, New Mexico, 87123, USA
[4] Department of Physics and Helsinki Institute of Physics, University of Helsinki, PO Box 43, FI-00014, Helsinki, Finland

Supplementary Results

Video of TEM tilt series. The attached video file Tilt_Series.mov is shown in Fig. S1 and presents the evolution of nanorod AuNR-7 under irradiation with 1.7 MeV Au ions as described in the main text and featured in Figs. 4 and S1.

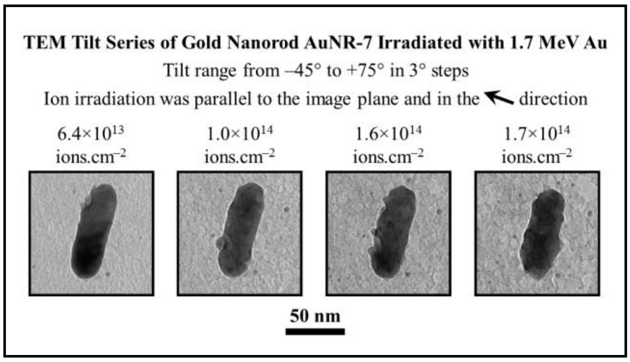

Figure S1. Still frame from attached video file Tilt_Series.mov showing the evolution of nanorod AuNR-7 under irradiation with 1.7 MeV Au ions.

Video of molecular dynamics simulation. The attached video file MD_Cascade.mov is shown in Fig. S2 and tracks the evolution of the cascade featured in Figs. 6(e–i).

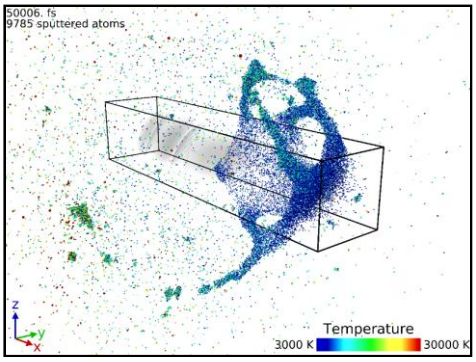

Figure S2. Still frame from attached video file MD_Cascade.mov showing the evolution of the cascade featured in Figs. 6(e–i).

Evolution of nanorods under 1.7 MeV Au ion irradiation. Table S1 shows the evolution of nanorods AuNR-1 to AuNR-6 under irradiation with 1.7 MeV Au ions as described in the main text.

Table S1. TEM image series of nanorods AuNR-1 to AuNR-6 under in situ irradiation with 1.7 MeV Au ions. As discussed in the main text, due to the tendency for the image analysis to overestimate the volume of particles with rougher surfaces, at low sputter yields it is possible for a negative value (i.e. an addition of atoms) to be calculated. The scale marker applies to all the micrographs in the table.

50 nm	**Ion fluence in xz-plane (×10^{15} ions.cm^{-2})**													**Sputter yield**
	**0.0**	**0.3**	**0.6**	**0.9**	**1.2**	**1.5**	**1.8**	**2.1**	**2.4**	**2.7**	**3.0**	**3.3**	**3.6**	
AuNR-1	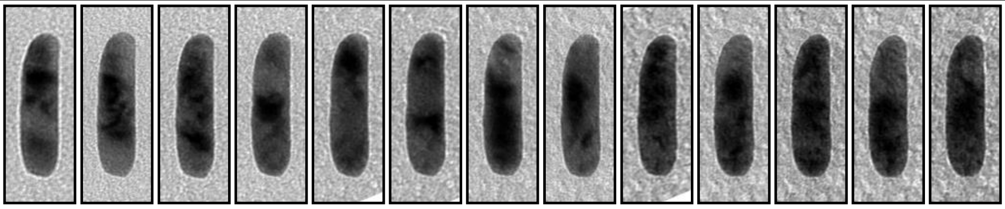													–2.1
AuNR-2	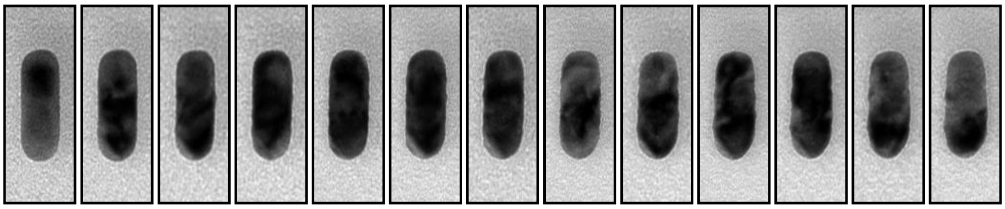													–1.0
AuNR-3	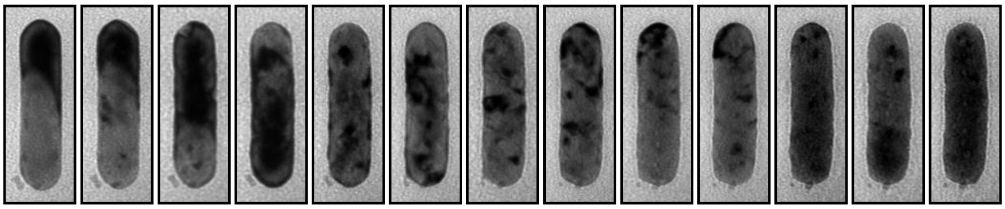													–0.34
AuNR-4	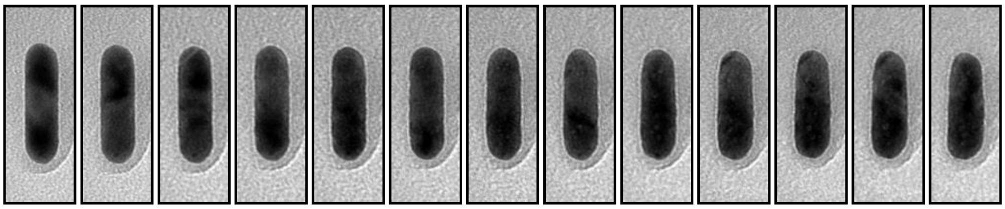													0.3
AuNR-5	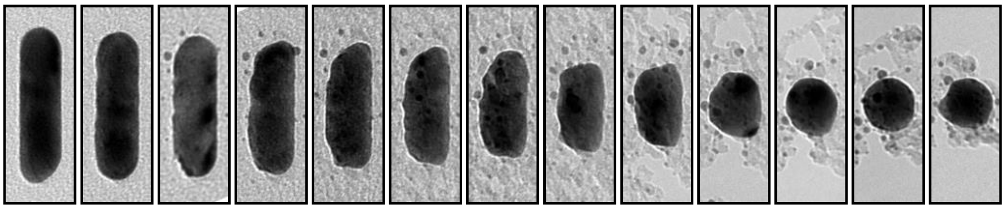													30.1
AuNR-6	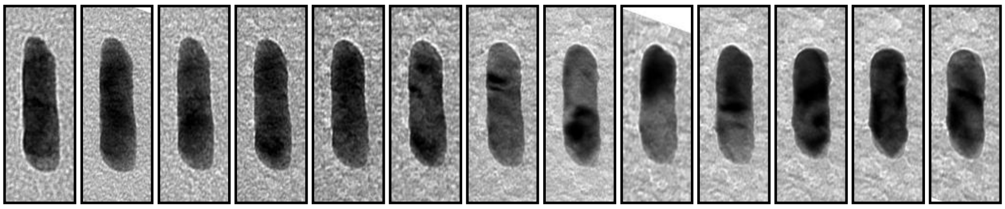													2.6

MDRANGE channelling map data file. The results of the MDRANGE channelling calculations plotted in Fig. 5 are included in the attached MDRANGE.csv file with the data arranged as detailed in Table S2. The data file contains the original results prior to the bilinear interpolation.

Table S2. Description of columns in the attached MDRANGE.csv file containing the results of the MDRANGE channelling calculations for 1.7 MeV Au ions incident on a 20 nm gold foil.

Column	Description
1	String – always “Res”
2	Polar angle, θ, in degrees
3	Azimuthal angle, φ, in degrees
4	String – always “f”
5	Fraction, f, of ions stopped (i.e. not reflected or transmitted)
6	String – always "+–"
7	Statistical error of f
8	String – always "R"
9	Mean range, R, of ions that stopped in the sample in Å
10	String – always "+–"
11	Statistical error of R
12	String – always "FDn"
13	Nuclear deposited energy, F_{D_n}, in the sample in eV
14	String – always "+–"
15	Statistical error of F_{D_n}
16	String – always "M"
17	h of ion direction written as a Miller-index-like vector
18	k of ion direction written as a Miller-index-like vector
19	l of ion direction written as a Miller-index-like vector

Supplementary Methods

Additional Details on TEM with in situ ion irradiation. A Hummingbird 1000 Series tomography holder was used for the TEM experiments in order to allow a wide range of x-tilts for investigation of the three-dimensional morphology of the nanorods before and after ion irradiation. As the TEM grid was suspended in free space in the mounting mechanism of the tomography holder, this choice of holder minimised the risks of ion or electron beam shadowing and of deposition of sputtered material from the holder onto the sample. To further reduce these risks, all samples were tilted about the TEM x-axis to form an angle of 45° with the ion beam during irradiation as shown in Fig. 1. For the experiments designed to investigate the effects of crystallographic orientation on the ion beam sputter yield, the samples were maintained at an x-tilt of +45° during both the irradiations and TEM imaging to avoid potential changes which may have been incurred by performing tilting operations.

The exception to this experimental methodology was nanorod AuNR-7 which was studied in a separate experiment designed to take greater advantage of the capabilities of the I^{3}TEM facility for real-time in situ observation. In that experiment, the electron beam was kept on during ion irradiation and a large number of nanorods were monitored until one was found which was exhibiting substantial changes due to the ion beam. The ion irradiation was then stopped and images were captured of that nanorod (AuNR-7) at x-tilts of –45° to +75° in 3° increments; the asymmetry in this angular range was due to the position of the nanorod relative to the mesh bars of the TEM grid and thus the extremes of tilt at which these caused electron beam shadowing. The ion irradiation was continued until further significant morphological changes had again occurred, irradiation was then paused and another tilt series was acquired. This procedure was repeated but was ultimately limited by degradation of the carbon support film under the electron beam and hence the end fluence was lower than for the experiments during which the electron beam was turned off during ion irradiation.

Determination of Crystallographic Orientation. As the TEM sample holder selected for these experiments had only a single-tilt capability it was not possible to manipulate the nanorods into perfect down-zone conditions in order to determine their crystallographic orientations. Therefore, selected-area diffraction patterns were captured of the nanorods at x-tilts starting from the initial orientation of the samples at 0° and then in +15° steps up to the irradiation orientation at +45°. A three-dimensional computer model was constructed featuring a FCC lattice centred at the intersection of the ion beam and the TEM electron beam which were normal to each other as per the geometry of the I^{3}TEM facility as shown schematically in Fig. 1. The model was allowed degrees of freedom to simulate the x-tilt and, initially, to allow the FCC lattice to rotate freely. Systematic rows of excited diffraction spots which were symmetric about the 000 were identified from the diffraction data for each nanorod. For each systematic row, the direction of the $\boldsymbol{g}$ vector in the xy-plane of the TEM and the x-tilt at which the reflections were excited were then used to define the orientation of the FCC lattice in the model. As is typical of many TEMs, the JEOL JEM-2100 in the I^{3}TEM facility features a small rotation between its image and diffraction modes as well as a 180° inversion; both of these were taken into account when incorporating the crystallographic directions from the diffraction data into the model. Once the orientation of the FCC lattice had been fixed, the model could then be viewed from the direction of the ion beam in order to investigate the crystallographic alignment of each nanorod relative to the incident ions and to measure the angles of tilt, θ, and twist, φ. Tilt, θ, is defined as the angle between the ion beam and the [100] direction and twist, φ, as the angle between the ion beam and the [001] direction projected onto the (100) plane. An example of this analysis is shown in Fig. S3.

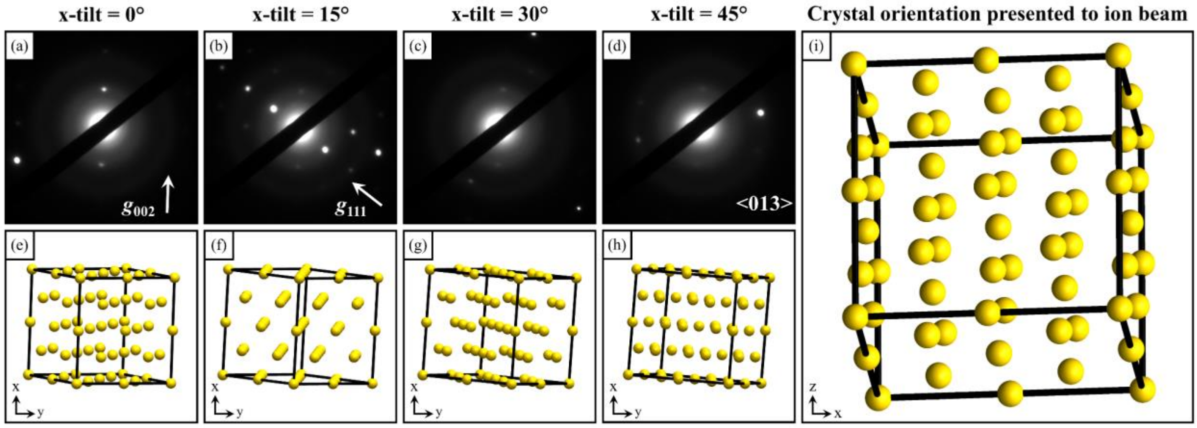

Figure S3. Example analysis of the diffraction patterns obtained from nanorod AuNR-5 to determine the crystallographic orientation presented to the ion beam during irradiation: **(a)** and **(e)** diffraction pattern featuring an assymetric row of 00*l* reflections and the corresponding orientation of the FCC lattice in the computer model at an x-tilt of 0°; **(b)** and **(f)** diffraction pattern featuring a 111 systematic row just off a <011> zone and the corresponding model at an x-tilt of +15°; **(c)** and **(g)** diffraction pattern and the corresponding model at an x-tilt of +30°; **(d)** and **(h)** diffraction pattern close to a <013> zone and the corresponding model at an x-tilt of +45°; and **(i)** the crystallographic orientation of the FCC lattice determined to have been presented to the ion beam during irradiation.

Calculation of Sputter Yields. In order to calculate the sputter yield per incident ion, N, for a given nanorod for each irradiation step, it is necessary to know the projected area, A, of the nanorod in the direction of the ion beam and the change in volume, ΔV, as a function of fluence step, $\Delta\Phi = 3\times10^{14}$ ions.cm^{-2}. The sputter yield for a single irradiation step is given by:

$$N = -\frac{\text{Sputtered atoms}}{\text{Incident ions}} = -\frac{\Delta V\ \rho}{\Delta\Phi\ A} \qquad \text{Equation S1}$$

Where ρ is the atomic density (ρ_{Au} = 59.03 atoms.nm^{-3}). The experimental sputter yields quoted are the averages of the yields for the individual irradiation steps.

Calculation of Number of Incident Ions. As the electron and ion beams form an angle of 90° in the I^{3}TEM facility and the x-tilt is normal to both beams, at an x-tilt of +45° the projected area of a nanorod is the same along both the z-axis (electron beam) and the y-axis (ion beam). Projected areas were measured by thresholding each image to isolate the featured nanorod from the background contrast of the carbon support film. The area of the resulting silhouette was then measured using the particle analysis function in the Fiji open-source image processing package[1]. An example of the image processing procedure is presented in Fig. S4.

As morphological changes were induced by the ion irradiation (during which the electron beam was switched off and thus direct observations was not possible), the projected area during an irradiation step was taken as the average of the projected areas of each nanorod before and after the irradiation step. The number of ions incident on a given nanorod during a given irradiation step was then calculated as this area, A, multiplied by the fluence step size, $\Delta\Phi$.

Calculation of Nanorod Volumes. To account for the varying diameters of the nanorods along their lengths, the silhouetted images of the nanorods were divided into short segments such that a typical nanorod of length 40 nm was cut into approximately 300 slices. The projected width of each segmented was then measured using Fiji. Each slice was assumed to be a thin cylinder and the volume of each nanorod before and after each irradiation step was thus calculated as the sum of the volumes of its slices. The volume change quoted for each nanorod is the sum of the 12 volume changes measured across the 12 irradiation steps. An example of the image processing procedure is presented in Fig. S4.

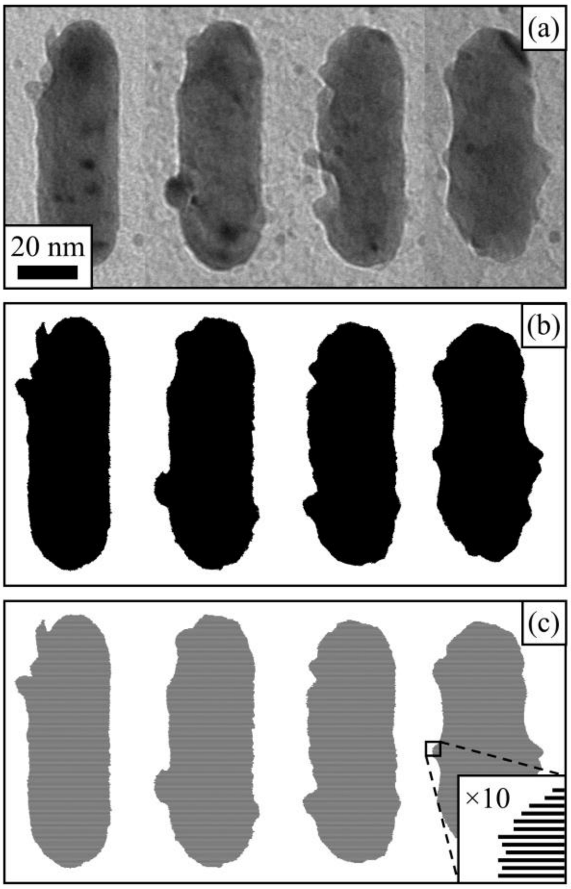

Figure S4. Image processing procedure by which nanorod projected areas and volumes were measured shown here for the example of AuNR-7 which features in Fig. 4 and the attached tilt series video: **(a)** a composite image comprising four micrographs captured after 1.7 MeV Au ion fluences of 0.6, 1.0, 1.6 and 1.7×10^{14} ions.cm^{-2} from left to right, respectively; **(b)** a binary image after thresholding to remove background leaving only silhouettes of the nanorod from which projected areas can be measured using Fiji; and **(c)** the silhouettes were divided into one-pixel-thick slices which were treated as a series of thin cylinders for which the individual volumes could be calculated and summed to give the total volume of the nanorod. The scale marker applies to all the images in the figure except for the magnified inset in **(c)**.

Ion Damage Profile Calculations. The damage profile for 1.7 MeV Au ion irradiation of a gold target was calculated using the *Stopping and Range of Ions in Matter* (SRIM[2]) Monte Carlo computer code version 2013. The code was run for 10^5 ions at normal incidence in the "Ion distribution and quick calculation of damage" mode with a displacement energy of 40 eV, lattice and surface energies of 0 eV, a target density of 19.30 g.cm^{-3} and a target depth of 250 nm. The phonon data output from SRIM was then used to generate the damage profile using the Norgett, Robinson and Torrens model[3] as per Stoller et al.[4]. The calculated damage profile in shown in Fig. S5.

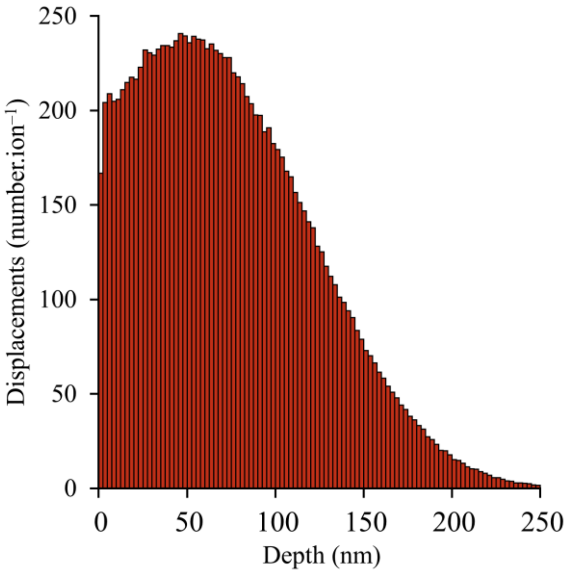

Figure S5. Damage profile for 1.7 MeV Au ion irradiation of gold calculated using the SRIM Monte Carlo computer code.

MDRANGE calculations on direction used for full molecular dynamics simulations. A statistical analysis has been performed on the nuclear energy loss calculated by MDRANGE along θ = 13° and φ = 0° (i.e. the direction used for the full molecular dynamics simulations). The results are presented in Fig. S6 and show that the most likely outcome is that there is practically no nuclear energy loss in the foil; in 50% of cases the nuclear energy loss was less than 2 keV. On the other hand, there is a clear peak at the maximum nuclear energy deposition at 1.65 MeV (the electronic energy loss was on average 53 keV). This corresponds to the Au ions that stop in the 20 nm foil and thus deposit all their energy into it. The former cases clearly correspond to zero or low, and the latter to the very high, sputtering in the full molecular dynamic simulations.

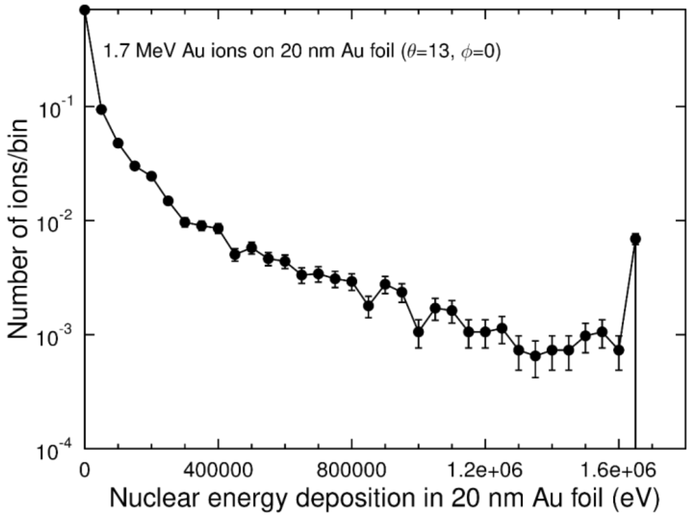

Figure S6. Statistics on the nuclear energy deposition by individual 1.7 MeV Au ions in a 20 nm Au foil obtained from the MDRANGE calculations for the case of θ = 13° and φ = 0° (i.e. the direction used for the full molecular dynamics simulations).

Supplementary References

1. Schindelin, J. *et al.* Fiji: an open-source platform for biological-image analysis. *Nat. Methods* **9**, 676–682 (2012).
2. Ziegler, J. F., Ziegler, M. D. & Biersack, J. P. SRIM – The stopping and range of ions in matter (2010). *Nucl. Instrum. Meth. B* **268**, 1818–1823 (2010).
3. Norgett, M. J., Robinson, M. T. & Torrens, I. M. A proposed method of calculating displacement dose rates. *Nucl. Eng. Des.* **33**, 50–54 (1975).
4. Stoller, R. E. et al. On the use of SRIM for computing radiation damage exposure. *Nucl. Instrum. Meth. B* **310**, 75–80 (2013).
